# Evaluating a Digital Health Tool Designed to Improve Low Sexual Desire in Women: Mixed-Methods Implementation Science Study

**DOI:** 10.2196/69828

**Published:** 2025-03-25

**Authors:** Lori A Brotto, Kyle R Stephenson, Nisha Marshall, Mariia Balvan, Yaroslava Okara, Elizabeth A Mahar

**Affiliations:** 1 Department of Obstetrics and Gynaecology University of British Columbia Vancouver, BC Canada; 2 School of Psychology Xavier University Cincinnati, OH United States; 3 Taras Shevchenko National University of Kyiv Kyiv Ukraine; 4 Department of Psychology State University of New York at Fredonia Fredonia, NY United States

**Keywords:** implementation science, sexual interest/arousal disorder (SIAD), sexuality, internet interventions, online therapy, telehealth, online interventions, web-based therapeutic programs/interventions, online CBT/MBT treatment, female sexual dysfunction, eHealth

## Abstract

**Background:**

Sexual health difficulties affect up to 30% of women, with desire and arousal problems being the most prevalent. While cognitive behavioral therapy and mindfulness-based therapy are effective treatments, access is limited by barriers such as specialist shortages, cost, and embarrassment. Web-based interventions offer a potential solution by providing self-paced, cost-effective treatments. eSense, a digital health program, offers cognitive behavioral therapy and mindfulness-based therapy skills targeted to women with low sexual desire, and previous trials find eSense to be highly feasible and efficacious.

**Objective:**

The goal of the present implementation science study was to use the RE-AIM (Reach, Effectiveness, Adoption, Implementation, and Maintenance of Implementation) framework to assess the integration of eSense into several sexual health clinics. We chose the RE-AIM framework because it addresses both dissemination (eg, reach) and implementation of an intervention.

**Methods:**

A total of 14 specialty clinics participated, and we report on the reported experiences of those clinics in implementing eSense. We also examined responses from 12 women on waitlists to receive sex therapy or sexual medicine care.

**Results:**

Per clinic outcomes, all aspects of implementation (reach, effectiveness, adoption, implementation, and maintenance) were in the moderate to high range for clinics, reporting that offering eSense helped them overcome negative feelings associated with their long clinic waitlists. The majority expressed a need for eSense and could see how it overcame the limitations of traditional therapy. Nearly all expressed a wish to continue offering eSense to patients after the implementation study was complete. One caveat was that half of the clinics noted cost as a key issue for future implementation, and one-third noted that the administrative burden of implementing eSense as a standard of care may be challenging. For individual users, the majority expressed an interest in knowing more about eSense and a desire to use eSense, though most of these did not complete the program in its entirety. Users experienced a significant improvement in sex-related distress with no clinically meaningful change in other outcomes and a high level of satisfaction with eSense. Most also reported doing things differently in their sexual lives after participating in eSense.

**Conclusions:**

We found that eSense demonstrates potential as a digital intervention for sexual difficulties for women, particularly concerning its moderate implementation outcomes and also because of its ability to reduce sexual distress. Future studies should address the barriers identified for broader adoption of eSense in clinical settings.

**Trial Registration:**

ClinicalTrials.gov NCT05168371; https://clinicaltrials.gov/study/NCT05168371

## Introduction

### Background

Difficulties with sexual function, including problems concerning orgasm, genital pain, desire, and arousal, are common and affect up to 30% of women [1]. Difficulties with desire and arousal are the most common [2] and are often triggered by a combination of biological, psychological, and sociocultural factors [3]. Difficulties with sexual arousal and desire can be distressing and lead to negative physical, emotional, and interpersonal outcomes, such as depressive symptoms and relational conflict [4-7].

Face-to-face cognitive behavioral therapy (CBT) and mindfulness-based therapy (MBT) are considered efficacious gold-standard treatments for desire and arousal difficulties in women [8-12]. CBT is change-based and aims to alter maladaptive thoughts and behaviors that impede healthy sexual response [13-15] and build new beliefs and behaviors that refocus attention to erotic cues and pleasure [16,17]. MBT is acceptance-based and encourages present-focused, nonjudgmental attention to body sensations [18,19]. Instead of changing specific thoughts and behaviors, MBT trains an individual to build interoception (ie, awareness of sensations in the body) and positive sexual awareness [20-22].

### Barriers to Accessing Treatment

Despite the prevalence of sexual problems and the existence of these evidence-based psychological approaches, there are system-level barriers to accessing help. These barriers include a lack of specialist providers, long waiting lists, high costs for private services, and discomfort talking about sex with professionals [23,24]. Another factor that might impact the willingness to seek help for sexual problems is the lack of awareness of available and evidence-based treatments [25,26].

One possible solution to these barriers is the use of web-based interventions. Here, we refer to “web-based interventions” as treatments that are fully digital rather than simply using internet technologies such as videoconferencing to support traditional face-to-face methods. These interventions are usually delivered online with varying degrees of synchronous or asynchronous guidance [27]. These interventions use digital technologies for screening, health promotion, prevention, early intervention, treatment, or relapse prevention [28].

Internet-based psychological interventions have been found to be as efficacious as face-to-face treatments in many cases [29]. Some advantages of web-based interventions are that they can be delivered at a self-determined pace and intensity, can maintain privacy or anonymity, and may be more cost-effective, potentially offering a more economically viable option for patients [30-33]. Additionally, because web-based psychological interventions are housed on accessible digital platforms, they are uniquely situated in their potential for scalability and widespread use. Of a systematic review investigating the quality of digital health interventions and among their list of 5 recommendations needed to establish that a digital tool is useful, one of them was that “the value delivered by digital health solutions should consider multiple dimensions such as clinical, organizational, behavioral, and technical” [34]. This includes ensuring that the intervention has benefits beyond just the clinical outcomes and benefits of the “system” in which treatments are delivered. As such, there is a need to evaluate the scalability of such interventions to determine their potential, including some of these multidimensional impacts on clinics that deliver care.

### Implementation of Web-Based Psychological Treatments

Implementation science involves expanding health care interventions beyond the controlled environment of evaluation studies into real-world contexts [35]. Due to randomized controlled trials often being criticized for limited generalizability [36], there is a growing need for implementation science to see how well effective treatments work when taken out of the strictly controlled research context and are delivered at scale. An interest in scaling up web-based psychological treatments has significantly increased in recent years [37] and has been well-received by many users of these services. For example, Guinart et al [38] found that 82% of patients who partook in a telepsychiatry appointment in the United States rated the overall experience as good or excellent, and 64% indicated they would consider using remote treatment sessions instead of face-to-face sessions in the future. Despite this growing interest, there has been limited research on the implementation of web-based psychological treatments related to sexual health concerns.

One of the few examples of an implementation science investigation in the domain of sexual health was the testing of a web-based program for sexual dysfunction in a Dutch public health setting. The study aimed to evaluate the implementation of a free, anonymous web-based education and self-training program for adolescents and young adults aged 16-24 years. The researchers were able to recruit over 1000 participants in the study but reported a significant dropout rate of 50%, with a primary contributor to the high attrition being the program’s substantial length [39]. The findings of studies such as this provide valuable insights into the facilitators and barriers to the implementation of digital sexual health interventions that we can integrate into future digital sexual health therapeutics. However, to our knowledge, there are no implementation science studies that have evaluated digital sexual health interventions specifically for adult women experiencing sexual difficulties.

### eSense: a Digital Sexual Health Tool

eSense is a web-based platform delivering psychological treatment of sexual health concerns that was originally designed to address sexual interest/arousal disorder [40] in females (though more recently it has been adapted to other populations of females with sexual concerns). This online intervention was created through a multidisciplinary collaboration among clinicians and researchers with expertise in sexual dysfunction, patient partners, web designers, and graphic illustrators. Individuals experiencing sexual difficulties often experience difficulty in accessing care due to geographic distance from qualified health care providers, cost, lack of health care providers specializing in sexual health, and emotional barriers (eg, anxiety, shame, or embarrassment) [41]; therefore, eSense may overcome these gaps and allow women with sexual interest/arousal disorder to access evidence-based care in the privacy of their homes. eSense is built on a WordPress PHP site (with Elementor plug-in) that is HIPAA (Health Insurance Portability and Accountability Act) compliant and uses a cloud server (Digital Ocean) based in Canada. It offers 2 distinct programs: CBT and MBT. Each program or “arm” consists of 8 modules that provide educational content, detailed instructions for therapeutic activities, fictional case examples to illustrate these activities, homework exercises, and troubleshooting tips. The intervention is delivered through a combination of text, video, images, and audio, all carefully designed to be highly engaging for users. Users have 2 options for completing the program: independently or with individual support from nonexpert “navigators” who offer empathetic listening, encouragement, and technology assistance but do not provide formal therapy [42]. Administrators from the research team create a unique log-in for eSense that is provided to an individual user following informed consent. Each user then has a unique database record associated with their account, and their engagement with eSense is then saved to their personal database for future analytics.

In a series of studies, eSense has been demonstrated to be usable and satisfactory, even without individualized guidance [35]. In a randomized trial evaluating eSense along with navigator support, participants reported significant and clinically meaningful improvements in sexual desire, sexual distress, sexual satisfaction, and overall sexual function compared to a control condition, with no loss in gains at the 6-month follow-up [42]. Taken together, while these studies found eSense to be feasible, satisfying, and efficacious when evaluated in the context of a controlled trial, its real-world implementation outcomes remain unknown, and there is a need to directly study unique factors associated with its implementation [43].

### Reach, Effectiveness, Adoption, Implementation, and Maintenance of Implementation Framework

Several implementation science frameworks facilitate the deployment of interventions at scale. Notable among these are the PRECEDE-PROCEED model, the Practical, Robust Implementation and Sustainability Model, the Consolidated Framework for Implementation Research, and the RE-AIM (Reach, Effectiveness, Adoption, Implementation, and Maintenance of Implementation) framework. The RE-AIM framework, in particular, is a widely recognized model in implementation science that covers five intervention-related areas of impact [44]: (1) reach as the proportion of those in need who access the intervention, (2) effectiveness per the impact of interventions on health outcomes, (3) adoption as a decision to proceed with implementing the clinical intervention, (4) implementation as the process of embedding and integration of the intervention in routine practice and its consistency of delivery and costs, and (5) maintenance as the institutionalization of the intervention in routine care [45-47]. This framework is considered more applicable than many other implementation science models, as it addresses both dissemination (eg, reach) and implementation aspects [48]. The RE-AIM framework provides a heuristic tool for bridging interventions’ internal validity established in well-controlled conditions and their external validity in real-world conditions [46,49]. It is designed to evaluate the public health impact of health-promoting interventions, and it is widely used in implementation research [44]. Glasgow et al [46,50] emphasize its utility in evaluating interventions that tackle multiple underlying factors and consider entire systems, and as such, may be ideal for evaluating the implementation of sexual health interventions. Of note, the hyphen in RE-AIM is intended to differentiate the individual-level factors (reach and effectiveness) from the organization-level factors (implementation and maintenance) [47]. One systematic review evaluating the RE-AIM framework concluded that all 5 domains should be reported on accurately and transparently to facilitate translation into real-world settings [47].

The overall goal of this study was to conduct an implementation science evaluation using the RE-AIM framework of the enablers and barriers to (1) patients using eSense when disseminated through sexual health clinics and (2) sexual health clinics implementing eSense into their existing structures. Due to the nonlinear, exploratory, and dynamic nature of implementation science research [35], we did not formulate a priori hypotheses. The findings were intended to also plan future implementation science studies with eSense once we implemented the key learnings about enhancing its implementation.

## Methods

### Research Design

We invited clinics in Canada and the United States to offer eSense to women who could not immediately receive treatment (ie, they were waitlisted at the clinic or they declined treatment at the clinic due to factors such as financial barriers). Using the components of RE-AIM [46], we sought to evaluate the impact of eSense by measuring its reach, effectiveness, adoption, implementation, and maintenance at the level of clinics as well as patients.

### Participants (Clinics and Patients)

Using the professional network of the first author, we identified several sexual medicine and sex therapy clinics in Canada and the United States and wrote to the clinic leads to inform them about this study. We used a combination of existing society membership listserves and word of mouth, as well as general internet searches to identify potential clinics that might participate. Those interested scheduled an information call with a research team member who explained this study and determined whether the clinic was eligible to participate and had the interest and capacity to refer women to this study. The eligibility criteria for the clinics were that they offered sexual health services and currently had a waitlist.

A point person at each participating clinic was provided with inclusion criteria for this study’s participants (ie, patients seeking care in their clinic). Women of any sexual orientation were eligible if they were aged 19 years or older, fluent in English, had no visual impairments, were computer literate with access to an internet-capable computer, had a history of sexual activity, and met telephone screening criteria for sexual concerns regarding low desire or arousal and associated distress.

### Procedure

Recruitment began in May 2022, and patients were referred to this study until December 2023. Each participating clinic invited females (either cis or trans women) who were seeking care in their clinic and were currently on their waitlist to consider participating in this study if they met the inclusion criteria (described above). Clinics collected basic information (ie, the reason for seeking treatment at the clinic, demographics, interest in participating in eSense) from all women who were contacted about participation in this study, regardless of whether those women went on to use eSense or not. This basic information was collected to compare participants versus nonparticipants of eSense*.* The women who participated were provided with the contact information for a member of the research team directly by the clinic staff person.

After consenting to this study, participants received a short description of each of the 2 arms of eSense (CBT and MBT) and chose one. If they did not have a preference, they were randomly assigned to an arm by this study’s coordinator.

Because prior research indicates that individualized support—even support that provides no formal therapy [51,52]—decreases attrition, increases adherence, and improves outcomes [53,54], participants had the option to have individualized support sessions with “navigators.” Navigators were undergraduate students who received training to encourage participants to engage in the treatment, and they answered practical questions [55]. Navigators were trained by one of the lead researchers on the eSense team. They were given a set reading list, which provided relevant background literature and provided with a standardized protocol and adherence documents for training. They then completed training sessions on the technical aspects of the role (eg, scheduling meetings) and on this study’s protocol and participated in an active listening skills workshop led by the researcher. Following this training, they participated in weekly group meetings and biweekly one-on-one meetings with this study’s coordinator. Participants could meet with a navigator as often as they wished (up to a maximum of once per week).

Participants filled out a baseline questionnaire using the online program Qualtrics and subsequently received access to the eSense website to work through one of the arms of the program at their own pace. Those who expressed interest in a navigator received a link to an online calendar that they used to schedule navigator meetings. Participants were sent their posttreatment questionnaire 1 week after their intended treatment end date (16 weeks from the date the participant first logged into eSense). At the end of this study, clinic directors and assisting clinic staff were also sent a questionnaire to access clinic-level implementation outcomes.

Because this study used an implementation approach with descriptive primary outcomes (eg, the proportion of women who were told about eSense and contacted us to express interest in participating and the proportion of women requesting navigator support), no power analyses were performed.

### Measures

Using the RE-AIM framework [46], we assessed the following outcomes:

### Clinic Level

1. Reach: the extent to which clinics were willing to participate in the implementation of eSense. Assessed by determining the proportion of clinics that were approached, expressed interest, and participated, including the reasons for their decisions.
2. Effectiveness: the extent to which clinics perceived eSense as impactful for their patients or the clinic. Assessed by gathering feedback from clinic staff on the effectiveness of eSense for their practice, including any observed benefits, as well as by determining whether user-level outcomes (eg, who was interested in eSense and who was not, participant satisfaction with treatment, or participant changes in sexual distress, interest, and desire) were meaningful to clinic partners (eg, does this information help them make decisions or achieve their goals).
3. Adoption: the extent to which clinics perceived the ease of adoption of eSense into their clinic. Assessed by determining if clinics had costs to support the maintenance of eSense and asking clinic staff why they chose to participate in this study using open-ended questions at the end of this study.
4. Implementation: the extent to which eSense was feasible to implement into their clinic. Assessed by determining the time that was required from research and clinic staff, resources required (if any), descriptions of adaptations, if any, made during this study (eg, changing the phone script at a specific clinic), reasons for adaptations (eg, to reduce costs or to have more people participate), and suggestions for improvements of the implementation procedure. Assessed by determining if clinics had costs to support the maintenance of eSense and asking clinic staff why they chose to participate in this study using open-ended questions at the end of this study.
5. Maintenance: the extent to which clinics perceived implementing eSense in their clinics as maintainable. Assessed by determining whether the organization would like to keep offering eSense, organizational barriers to offering eSense, reasons for continuance or discontinuance, and whether the clinic was willing and able to pay for potential costs associated with distributing eSense.

Clinic feedback forms also included options to provide free responses to any of the questions that also requested quantitative data.

### Participant Measures

1. Reach: the extent to which participants were willing to participate in eSense. Assessed by determining the characteristics of those who participated in the intervention versus those who did not and why.
2. Effectiveness: the extent to which participants benefited from eSense. Assessed by examining changes from baseline to posttreatment in (1) sexual distress, measured by the Female Sexual Distress Scale-Revised [56] and (2) sexual desire, measured by the Sexual Interest and Desire Inventory [57]. As recommended by RE-AIM [46], we measured change in satisfaction with life [58] and satisfaction with sex life. We also assessed satisfaction with treatment by an adapted version of the Erectile Dysfunction Inventory of Treatment Satisfaction Scale [59] and attrition rates.
3. Adoption: the extent to which participants were willing to start eSense. Assessed by determining hesitations participants had before joining this study and starting eSense and if participants were engaged in any other treatment while using eSense.
4. Implementation: the extent to which participants engaged with eSense. Assessed by determining how participants used the intervention, including the average number of modules completed and reported barriers to engaging with eSense.
5. Maintenance: the extent to which participants perceived the long-term effects of eSense. Assessed by determining whether participants planned to continue practicing eSense homework in the future.

Additionally, we examined the demand for navigator support, preference for MBT versus CBT, changes from baseline to posttreatment in motivation to see a health care provider for their sexual concerns, romantic partner involvement, and whether the participants dropped from the clinic waitlists after eSense participation.

### Analysis Plan

For our quantitative effectiveness outcomes, we used a paired samples *t* test with 1 within-subject factor of time (treatment) and 2 measurement points (baseline and posttreatment) and included effect sizes, as measured by Cohen *d*. We analyzed the qualitative data descriptively with a focus on key themes.

### Ethical Considerations

This study was approved by the Behavioural Research Ethics Board at the University of British Columbia (certificate #H22-02993), and all participants provided written informed consent. All clinic and individual participant data were deidentified before analyses, and all data were analyzed as a group. Although participants were not compensated for participation, clinic-level administrative staff who assisted with data collection were compensated for their time at the hourly rate paid for by their clinics.

## Results

### Clinic Results

#### Reach

Of the 194 clinics contacted, 178 did not respond, and 16 wanted to learn more about this study. Of these, 14 (7.2% of the total contacted) clinics agreed to participate after learning more about the details and requirements of this study.

Most clinics involved in this study provided psychological treatment of sexual difficulties (n=13, 92.9%), with fewer offering physical therapy or pharmacological treatment (n=4, 28.6%). A total of 13 of 14 clinics specialized in the treatment of sexual difficulties. In free-response questions at the end of participation, clinics indicated that the most common reason for referring women to this study was because they presented with low desire, arousal issues, or painful intercourse. Clinics also reported that they most commonly referred younger women and women with financial constraints.

#### Effectiveness

Per effectiveness, the outcomes collected in this study were meaningful to 71.4% (n=10) of clinics. Clinics reported that this study provided valuable support in decision-making, particularly in determining whether referring clients to similar studies would be beneficial. Additionally, the information collected helped shape organizational decisions such as business plan adjustments, next steps for possibly embedding eSense into their clinic, guiding offerings to clients, and improving the services. Some remarked:

Super important information that can help direct our business plan, next steps, guide offerings to clients.

Would be interested in all that information. That would be helpful in deciding how to utilize the program.

Per clinics’ motivation to participate in this study, most reported their desire to support research, to provide additional client support for those on the waitlist, and to help explore the use of eSense in clinic operations. Several clinics mentioned that being able to offer eSense to waitlist patients alleviated some of the provider guilt associated with extensive waitlist times.

#### Adoption

Responses regarding adoption focused largely on budget considerations. Regarding the budget for maintaining eSense, 50% (n=7) of clinics stated that their ability to offer eSense to patients depended on its cost, while 28.6% (n=4) indicated they could not support the maintenance costs. One of the clinics stated:

We wouldn't be able to afford supporting a cost for clients without getting some reimbursement from clients. If clients were choosing to enroll, and paying for the program directly, we could absolutely support it. We do not have the funds to sponsor it for our clients.

Sexual medicine center

The majority, 78.6% (n=11) of clinics, expressed a need for eSense in their clinic, citing the limitations of traditional therapy sessions and the importance of providing clients with additional support for practicing therapeutic tools. One remarked:

Therapy is often the space we are processing emotional content. It is nice to provide options for clients who want to have support practicing tools.

#### Implementation

Regarding implementation, feedback from clinics revealed a strong interest in continuing to offer eSense after this study, with 92.9% (n=13) of clinics indicating their willingness to do so. The primary reasons for this interest included the program’s ability to complement traditional 1-hour therapy sessions by providing additional tools and resources, thereby offering clients more support options. Clinics also highlighted the perceived value of eSense as a resource for women who lack access to sex therapy, noting its reliability and creation by experts in the field. Some of the clinic responses included:

Only so much can be covered in a typical one-hour therapy session. Providing clients with tools and resources is of utmost importance to me and my practice.

It's a very valuable resource, and much needed! Plus it is offered by the experts in the field, so it is a reliable source.

Another option for women to do in their own time and in the comfort of their own home. Seems like a great option for clients who either can't afford therapy, or who are stuck on a wait list.

However, 35.7% (n=5) identified barriers to continuing eSense, primarily related to resource intensiveness (eg, additional time required from clinic intake coordinators who would refer women to the program and costs associated with this). Additionally, although clinics were required to have a client waitlist to be eligible for this study, many clinics did not end up having waitlists at their clinics, which reduced their immediate need for eSense. Despite this, they acknowledged that eSense would be valuable when immediate treatment was not possible. There were also challenges in keeping triage and care management staff updated about the program, which would require additional training to keep staff informed about eSense. As 1 clinic remarked:

The only barrier is that we've been able to offer timely treatment to potential clients, so clients did not have a need to take part in eSense. However, we realize timely treatment may not always be possible. So it's great to have eSense as an option.

#### Maintenance

Regarding maintenance, 92.9% (n=13) of clinics believed that eSense could be successfully integrated into existing clinic operations. Clinics suggested various methods for integration, including using eSense as a support outside of the office settings, assisting with take-home practices and self-paced work, and including eSense as part of a stepped-care model. They also saw the potential for eSense to be an adjunct to ongoing therapy and a resource for current patients, in addition to patients placed on the waitlist. Clinics indicated the possibility of being a referral platform by adding eSense to their resource information lists and websites. In response to asking how eSense could be integrated into the framework of their organization, some of the clinics remarked:

It would be part of a stepped-care model that all clients are assessed for.

Patients receive a variety of resource information and eSense would be added to this list. Providers can continue to speak about its benefits.

I'm not sure yet. But one thing that might be possible is that we could offer a link to eSense on our website in the areas about desire and arousal concerns -- we have a section for each concern with ideas for resources to try on one's own. As long at [sic] the tool is not costly, we could also offer it as we have been for people who choose not to enroll in sex therapy due to cost or other factors.

### Participant Results

#### Reach

**Figure 1:**
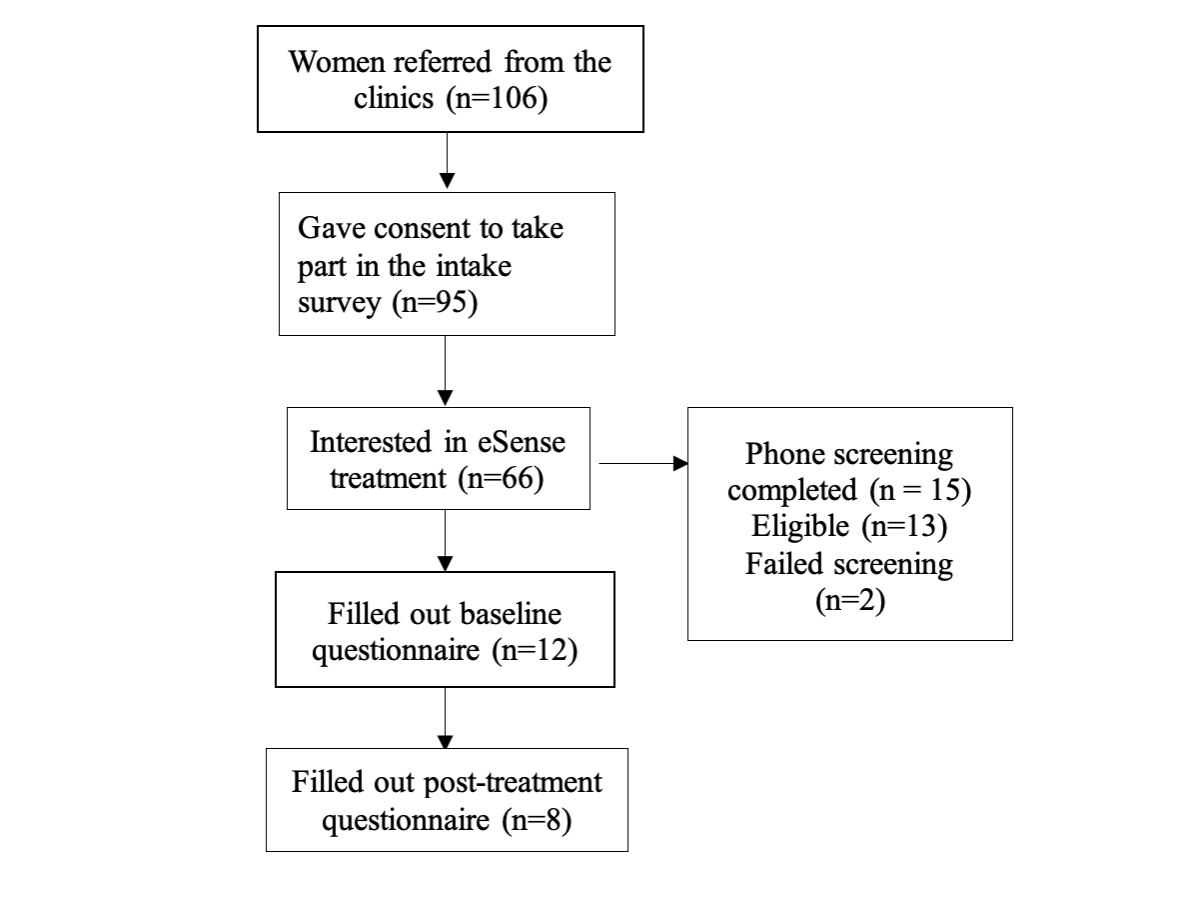
Diagram of clinic and participant flow from referral through all stages of participation.

Regarding how well we were able to reach patients, 14 partner clinics referred a total of 106 women to our study, and of those, 95 provided consent to take part in the intake survey and fully or partially filled it in (Figure 1).

Of the participants who reported why they were seeking treatment at the participating clinic (n=81), nearly half (n=40, 49.4%) indicated that this was for a sexual desire or arousal concern, while the remaining 50.6% (n=41) were seeking help for various other issues such as mood and anxiety, sexual trauma, menopause symptoms, hormone imbalances, and other health concerns.

Among those seeking treatment at the clinic, 39 (55.7% of 80 responses) participants were informed they would be placed on a waitlist for treatment at that clinic. A total of 51 (79.7%) planned to proceed with treatment from their referral clinic, whereas 13 (20.3%) decided to no longer seek treatment from the clinic. The reasons for not seeking treatment included wait times, the transition to primarily online counseling, financial constraints, and insurance coverage issues.

After being told about eSense, 66 (82.5%) women expressed interest in learning more about it, while 14 (17.5%) were not interested in using eSense (Figure 1). Reasons for lack of interest included a perceived irrelevance to their situation, lack of time, preference for in-person interactions, not understanding the treatment, and reporting being already satisfied with their current sex life.

Of the 66 who initially expressed an interest in this study, only 12 went on to participate in this study, and all identified as cisgender (Table 1). Those who decided to participate provided various motivations, such as ongoing issues with arousal difficulties, low desire, and sexual anxiety, which had made participating in sex stressful and guilt-inducing. Other reasons for participating included severe body image issues, histories of eating disorders, and the resulting body shame that inhibited their sexual lives. Others expressed distress over a lack of physical attraction to their primary partners despite emotional connections, leading to relationship stress. A common theme was a marked decrease in libido and a desire to explore new concepts of attraction and improve their sexual lives.

Per reasons for the high rate of attrition, we compared participants to nonparticipants on select demographic variables that were collected among those who declined participation. The 2 groups did not differ in age (*P*=.91), race (*P*=.80), relationship status (*P*=.41), or sexual orientation (*P=*.054).

**Table 1:** Participant demographic information at baseline (N=12).

Characteristics		Values
Age (years), mean (SD)		40 (11)
**Gender identity,** **n (%)**		
	Woman	12 (100)
**Sexual orientation,** **n (%)**		
	Bisexual	4 (33.3)
	Heterosexual	7 (58.3)
	Pansexual	1 (8.3)
**Racial identity,** **n (%)**		
	Racialized, person of color, visible minority, or non-White	1 (8.3)
	Mixed or biracial	1 (8.3)
	Mix-White or non-White	1 (8.3)
	White, European descent	9 (75)
**Education, n (%)**		
	Attended some college or university	1 (8.3)
	Graduated 2-year college or university	2 (16.7)
	Graduated 4-year college or university	3 (25)
	Postgraduate degree	6 (50)
**Annual household income (in CAD $^a^), n (%)**		
	$40,000 to $59,999	1 (8.3)
	$80,000 to $99,999	3 (25)
	$100,000 to $159,999	3 (25)
	$200,000 to $239,999	3 (25)
	More than $300,000	1 (8.3)
	Prefer not to answer	1 (8.3)
**Employment status, n (%)**		
	Full time	8 (66.7)
	Part-time	1 (8.3)
	On disability	1 (8.3)
	Freelance	1 (8.3)
	Other	1 (8.3)
**Religious affiliation, n (%)**		
	Christian-Protestant	3 (27.3)
	No religious affiliation	8 (72.7)

^a^A currency exchange rate of CAD $1=US $1.45 was applicable.

#### Effectiveness

Data for effectiveness are presented in Table 2 and is based on 8 participants who provided full pre and post data. A series of paired samples *t* tests evaluated the effects of eSense on clinical outcomes. Sexual desire (as measured by the Sexual Interest and Desire Inventory) did not significantly differ from pretreatment to posttreatment (*P*=.94), with a negligible effect size (Cohen *d*=0.029), indicating no meaningful change in sexual desire after eSense. The total Female Sexual Distress Scale-Revised score, however, decreased significantly from pretreatment to posttreatment (*P*=.004), with a large effect size (*d*=–1.459), reflecting a substantial reduction in sexual distress. Satisfaction with life did not significantly change from pre- to posttreatment (*P*=.31), with a small effect size (*d*=–0.388). Similarly, satisfaction with sex life also did not significantly change from pre- to posttreatment (*P*=.31), with a small effect size (*d*=–0.388).

**Table 2:** Pretreatment and posttreatment measures of sexual desire (SIDI^a^), sexual distress (FSDS-R^b^), satisfaction with life (SWLS^c^), and satisfaction with sex life (SWSL^d^). Data presented are means and SDs based on 8 participants who provided full data.

Variable	Pretreatment, mean (SD)	Posttreatment, mean (SD)	Cohen *d*	*P* value
Sexual desire (SIDI)	22.29 (8.98)	22.43 (9.4)	0.029	.94
Sexual distress (FSDS-R)	32 (10.74)	24.88 (10.97)	–1.459	.004
Satisfaction with life (SWLS)	28.63 (5.88)	27.25 (5.01)	–0.388	.31
Satisfaction with sex life (SWSL)	22.9 (4.7)	21.8 (4.01)	–0.388	.31

^a^SIDI: Sexual Interest and Desire Inventory.

^b^FSDS-R: Female Sexual Distress Scale-Revised.

^c^SWLS: Satisfaction With Life Scale.

^d^SWSL: Satisfaction With Sex Life Scale.

Participants’ satisfaction with eSense was evaluated in various ways. We measured satisfaction with the experience of having versus not having a treatment navigator. Participants were evenly divided in their initial choice to have the support of a treatment navigator before starting eSense, with 4 choosing to have a navigator and 4 opting not to. Across those who chose to work with a navigator, the total number of navigator sessions was 12. Those who opted for the support of a treatment navigator expressed a range of opinions. Some appreciated having someone to discuss their thoughts and feelings with, which helped them process information and feel validated. Others felt that scheduling meetings and clarity of the navigator’s role could have been better. Overall, the mean satisfaction score regarding participants’ choice to have a navigator corresponded with “somewhat satisfied.” Some felt they would have been more dedicated to the program with a navigator’s support but believed they could manage on their own due to their previous therapy experiences. Others preferred not to discuss their concerns with anyone who was not a fully licensed therapist.

Participants provided some feedback on barriers that arose that prevented them from fully engaging with eSense*.* These included no time due to their job and taking care of children, lack of privacy, avoiding the exercises, and feeling frustrated that a partner was not involved in trying to improve the sexual issues.

The overall mean Erectile Dysfunction Inventory of Treatment Satisfaction Scale score was 73.64 (SD 14.85) of 100, suggesting a high level of satisfaction with eSense*.*

#### Adoption

One-third of participants (4 of 12) were initially hesitant to join this study, mainly due to concerns about the time commitment required. The average wait time for participants before they were likely to see a provider was 17 weeks, and many of them agreed to participate in eSense to address their concerns on the recommendation of health care providers or as a proactive step before starting therapy. Most participants were not engaged in any other treatment at the time, although a few mentioned ongoing treatments for other health issues (eg, thyroid medication or pelvic floor physiotherapy).

#### Implementation

When given the choice of which arm to use, 6 initially requested CBT, and one of these requested a change to mindfulness after starting. Further, 5 participants requested mindfulness from the outset, and 1 participant did not express a preference. Of the 12 who started eSense*,* 8 completed posttreatment measures, with only 37.5% (3/8) of these completing all 8 modules. The average number of modules completed was 4.3 (of 8). Engagement with homework activities also varied: 25% (n=2) did none, 12.5% (n=1) did a few, 37.5% (n=3) completed about half, and 25% (n=2) engaged with all the homework activities. Barriers to consistent homework engagement were reported by 87.5% (n=7) of participants.

Regarding barriers to engaging with eSense, participants cited various personal and situational challenges. These included changes in job responsibilities, medical issues such as low iron causing fatigue, lack of time and privacy, mental blocks, and frustration from the perception that they were attempting to address a problem primarily viewed as significant by their partner rather than by themselves. Despite these challenges, a majority (87.5%) of participants reported doing things differently in their sexual lives as a result of eSense. These changes included letting go of guilt, practicing mindfulness, improving self-talk, and increasing communication in the bedroom. Some participants also noted changes in the nonsexual aspects of their lives, such as increased mindfulness and attunement to pleasure.

#### Maintenance

All participants indicated they would continue to use what they learned from eSense in the future, such as the new mindfulness practices, journaling, and the use of “sexual aids” (ie, vibrators, erotica, and fantasy). Participants highlighted the importance of awareness of thought patterns and cognitive biases, with some intending to reflect on these and try behavioral experiments to overcome them. Even those who did not progress far into the material found value in the initial modules and planned to incorporate those learnings into their lives.

#### Additional Findings

Participants reported increased awareness and a sense of empowerment regarding their sexual health. However, suggestions for improving the program included the addition of more videos, more time between modules, greater clarity on certain instructions, and more flexible options for engaging with a navigator.

## Discussion

### Principal Findings

This study aimed to evaluate the implementation of eSense, a digital therapeutic intervention including CBT and MBT, for women experiencing sexual difficulties and seeking care from specialty sexual health clinics. Using the RE-AIM framework, we assessed eSense’s reach, effectiveness, adoption, implementation, and maintenance across clinic and participant levels. Overall, eSense was associated with a meaningful reduction in sexual distress among participants, with moderate adoption by clinics and high satisfaction from both clinics and participants, despite some limitations in engagement and completion rates.

At the clinic level, a significant challenge was reaching clinics that would be willing to adopt eSense. Although 194 clinics were contacted, only 14 ultimately participated, and many of the nonparticipating clinics indicated that their waitlist was very short and patients seeking treatment were able to be seen quickly by a provider. Many clinics expressed interest in offering eSense as additional support for their waitlisted patients, which alleviated some of the strain associated with long wait times. This finding aligns with prior research suggesting that digital interventions can complement traditional care models and offer value to health care providers in overburdened systems [52].

However, clinics also cited resource limitations, including staff time and financial constraints, as barriers to long-term adoption. This finding is consistent with a systematic review of 221 eHealth intervention studies focused on the barriers and facilitators of successful outcomes [60], which found that an increase in workload and workflow disruption posed significant barriers to implementing eHealth tools [60]. Such barriers are likely particularly important for integrating digital health tools in smaller, less-resourced clinics [54] and, given that most partnering clinics in this study offered psychological services, they may be smaller or less well-funded (or not publicly funded at all) in comparison to larger medical centers.

These findings make it important to explore alternative methods of implementing eSense, including different treatment settings and varying levels of integration. For example, the PTSD Coach App was created to translate gold-standard psychotherapy for posttraumatic stress disorder into a self-guided or clinician-aided online tool similar to eSense. While PTSD Coach is often used by providers in specialty trauma clinics [61], it has also been successfully integrated into generalist primary care clinics, both with and without provider support [62]. One of the benefits of such an approach is that it is possible to achieve broad reach without consistent formal implementation work because the program is viewed as a stand-alone tool rather than a new labor-intensive addition to clinic procedures [63]. Similarly, eSense might achieve maximum reach by being an external resource recommended in general treatment settings rather than via integration into specialty care.

However, existing as a stand-alone tool raises the question of who will or can pay for the service. Indeed, the cost came up during our assessment of adoption, with several clinics noting that they would not be able to cover the additional costs incurred from the use of eSense in the future. During the present trial, eSense was evaluated within a research context, and as such, there were no costs to the clinics that adopted it. However, in the future, when eSense is incorporated and commercial, there may be necessary costs to clinics associated with leasing or purchasing a license. Alternatively, end users could pay for eSense access, with clinics serving only as referral sources. While the specific costs of such activities to clinics or users are unknown at the moment, anecdotal feedback to our team from potential users suggests that those residing in the United States were more likely to expect eSense to be covered by a third-party payer, whereas Canadian potential users were open to paying an individual subscription fee.

Who should bear the cost of interventions such as eSense is fundamentally an issue of equity, and if the cost is shouldered by end users, many of the same inequities present in traditional health care may be replicated, with historically underserved groups having less access and experiencing greater burden. To avoid such negative outcomes, a 2022 summit of the Agency for Healthcare Research and Quality provided a set of recommendations [64] that emphasized the importance of primary care as an avenue of equitable implementation of care, along with the need to actively engage with members of underserved communities, and partner with culturally relevant sectors such as faith organizations and tribal communities. In line with such recommendations, future implementation of eSense should (1) identify ways in which care settings can budget for the additional time and costs associated with integrating it into clinical operations, (2) include active engagement with members of underserved populations (eg, sexual and gender minority individuals), or (3) explore the implementation of eSense in a variety of settings that can allow for equitable access.

At the participant level, eSense was well-received by participants who engaged with it, as evidenced by high satisfaction scores. Of note, of 66 women who initially expressed an interest in this study, only 12 participated and provided pre-post questionnaire data sufficient for analysis. This small sample size impacted the power of our statistical analyses, and as such, the effect sizes obtained should be taken as preliminary. Although 87.5% of participants did not complete all 8 modules, there was a significant reduction in sexual distress and a willingness to continue practicing the skills learned in eSense beyond this study. These findings are consistent with the PLISSIT (Permission, Limited Information, Specific Suggestions, and Intensive Therapy) model [65], which suggests that many individuals with sexual concerns can experience meaningful benefits from early interventions such as normalization and permission-giving elements introduced in eSense’s first module. Therefore, the low completion rates may not necessarily reflect dissatisfaction or ineffectiveness but rather that participants received sufficient benefit from earlier parts of the program. The minimally effective dose of eSense to lead to meaningful clinical improvements is unknown and should be the focus of future research.

Although eSense was efficacious in this trial and the original randomized trial [42], this efficacy was not sufficient for successful implementation. It is well documented that the factors that contribute to a treatment being efficacious in a controlled research study are fundamentally different from programs that are successfully implemented [66]. One reason for the difference is that efficacy trials have strict rules on the setting, conditions, participants, and other factors, whereas implementation trials must be robust across a variety of different settings, conditions, and participants [42]. Another reason is that efficacy trials attempt to control variance by restricting the conditions, whereas implementation trials seek to understand variation across different settings. Given that eSense was highly efficacious but challenges arose with implementation, this suggests that there is a need to understand the moderating factors that contribute to its successful implementation in the future, as these moderating factors will be important if eSense is to be successfully implemented.

Our findings imply that digital interventions such as eSense can be a valuable supplement to traditional therapy, particularly for individuals facing barriers to accessing care, such as long wait times or financial constraints. eSense’s ability to reduce sexual distress supports the notion that digital tools may fill critical gaps in the health care system by offering low-cost, accessible solutions, and even without improving key symptoms, the reduction in distress may be particularly meaningful for patients to increase their readiness for future interventions. However, this study also highlights the importance of “fit” when introducing such interventions. Women seeking specialty care at sexual health clinics may differ significantly from the general population, having already invested substantial time and resources into accessing specialized care. These individuals may be less likely to engage fully with digital interventions such as eSense, especially if their expectation is face-to-face therapy.

Despite the promising results of eSense’s implementation, this study has several limitations. The primary limitation is the relatively small number of participants, which restricts the generalizability of the findings. Additionally, because we did not formally capture user engagement data (apart from homework completion), we do not know how much participants actually engaged with eSense and what dose of the treatment they were exposed to. There is also a need to implement structured checklists specific to digital health interventions to determine their value [34]. Finally, most participants did not work with a navigator as they completed the eSense modules. Given that the randomized controlled trial showed excellent efficacy when participants were assigned a navigator [42], it is possible that any future implementation of eSense may be more successful if navigator support is included.

### Conclusion

This paper addressed a significant gap in the literature on enablers to implement effective sexual health treatments in the clinical setting. This study found that eSense demonstrates potential as a digital intervention for sexual difficulties for women, particularly concerning its moderate implementation outcomes and also because of its ability to reduce sexual distress. However, challenges related to clinic adoption and participant engagement suggest that future iterations may need to consider scalability, clinic resource constraints, and the specific needs of participants seeking specialized care to increase their engagement. In particular, we recommend that costs to clinics be kept minimal, that methods of reducing attrition and enhancing adherence to modules and homework for users be considered, and that flexible options for engaging with navigators might be considered in future implementations of eSense*.*

## Abbreviations

CBT: cognitive behavioral therapy
HIPAA: Health Insurance Portability and Accountability Act
MBT: mindfulness-based therapy
PLISSIT: Permission, Limited Information, Specific Suggestions, and Intensive Therapy
RE-AIM: Reach, Effectiveness, Adoption, Implementation, and Maintenance of Implementation

## Data Availability

The dataset for this study is available at Open Science Framework [67].
